# Supplementary material for: Spatial and temporal intratumour heterogeneity has potential consequences for single biopsy-based neuroblastoma treatment decisions
Source: Nat Commun. 2021 Nov 23;12:6804. doi: 10.1038/s41467-021-26870-z (PMC8611017; doi:10.1038/s41467-021-26870-z)
Supplement: Supplementary file 1 — Supplementary Information [file 41467_2021_26870_MOESM1_ESM.pdf]

Supplementary Information for Schmelz et al. *"Spatial and temporal intratumour heterogeneity has potential consequences for single biopsy-based neuroblastoma treatment decisions"*

Supplementary Figures

- Supplementary Figure 1: Overview of intratumour heterogeneity of SNVs analysed by WES and ultra-deep targeted sequencing across the cohort
- Supplementary Figure 2: Intratumour heterogeneity of SNVs analysed by ultra-deep targeted sequencing across the cohort
- Supplementary Figure 3: Validation of the temporal heterogeneity of the *ALK* R1275Q mutation in samples from CB1008 using digital droplet PCR (ddPCR).
- Supplementary Figure 4: Comparison of phylogenetic evolution based on SNVs and SCNAs
- Supplementary Figure 5: *MYCN* FISH analysis and SNP array confirm temporal ITH of MNA in samples from patient CB1003.
- Supplementary Figure 6: Temporal and spatial changes in RNA expression assessed by multi-region RNA sequencing in consequence of SCNA

Supplementary Tables

- Supplementary Table 1: Patients characteristics
- Supplementary Table 2: SNVs in cancer-related genes and their biological relevance

Supplementary References

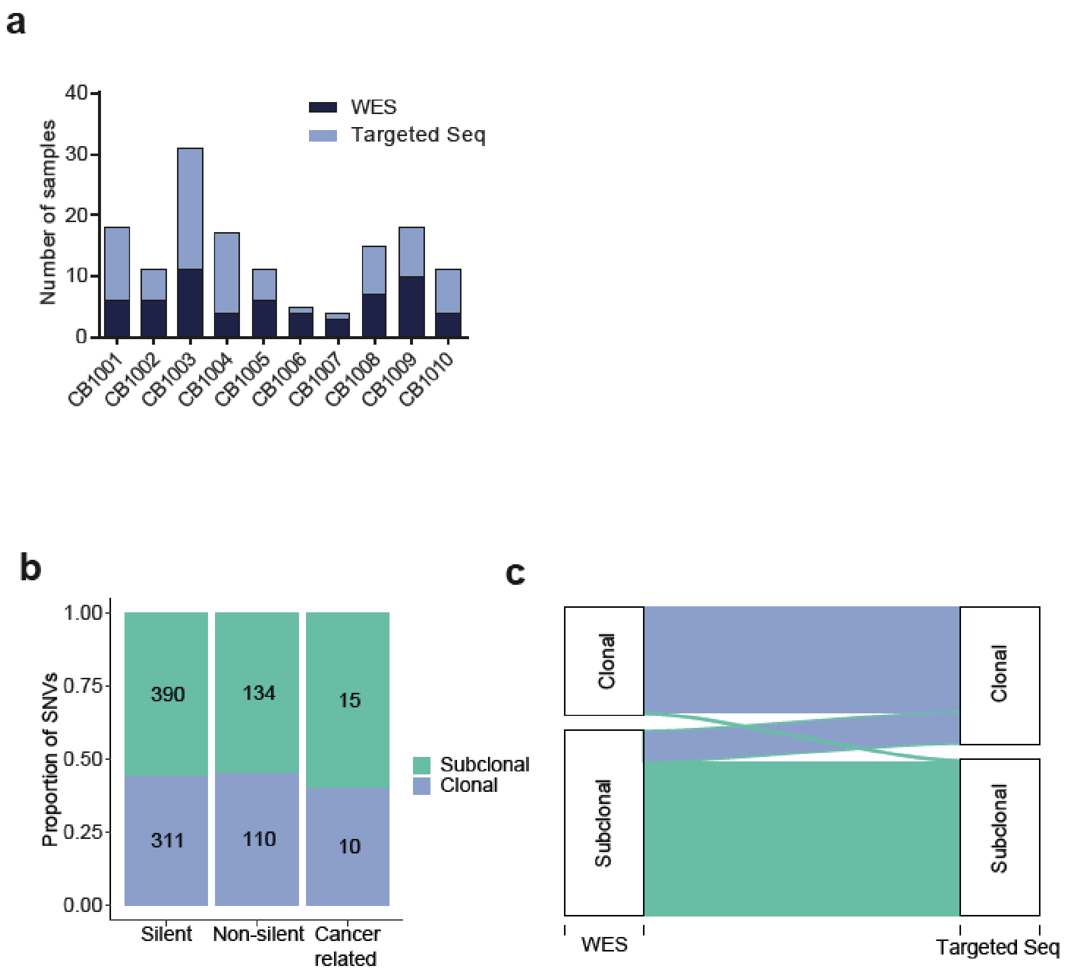

Supplementary Figure 1: Overview of intratumour heterogeneity of SNVs analysed by WES and ultra-deep targeted sequencing across the cohort. a Number of tumour samples per patient analysed by WES and targeted sequencing. **b** Bar plot showing the ratio of clonal to subclonal presence of silent, non-silent or cancer-related SNVs. Silent SNVs refer to either intergenic, intronic or synonymous SNVs that do not create changes in the amino acid sequence of the translated protein. Non-silent refers to SNVs within exonic regions that cause an altered amino acid sequence. The category "cancer-related" indicates non-silent SNVs within any of the 801 genes considered to be cancer-related according to COSMIC database and literature (see methods). **c** Alluvial plot comparing the categorization of SNVs between WES (WES) and targeted amplicon sequencing (targeted seq) data. SNVs with a variant allele frequency (VAF) >10% in all samples in WES or targeted sequencing data were defined as "clonal". All other detected SNVs were considered subclonal.

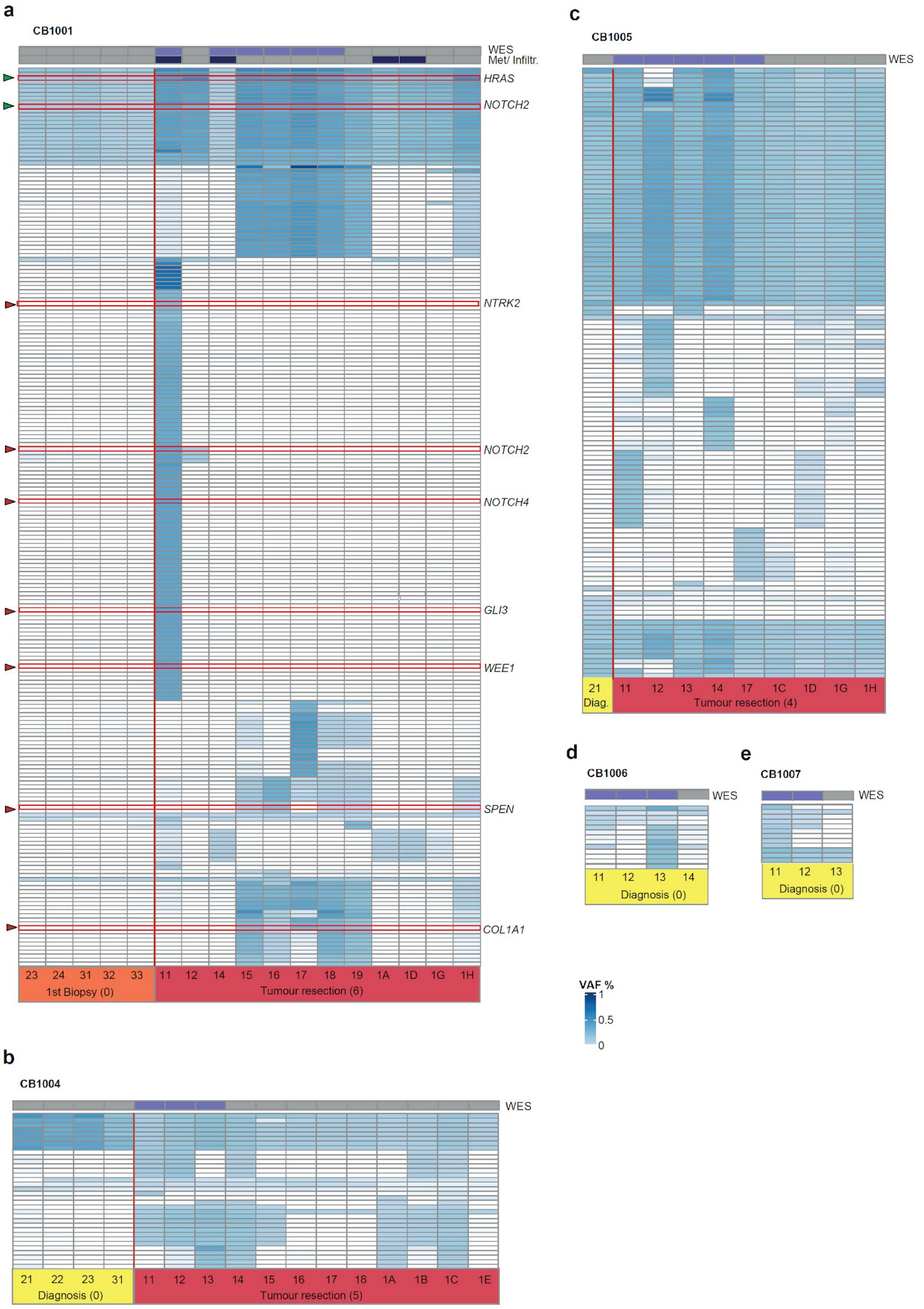

a
CB1001
WES
Met/ Infiltr.
HRAS
NOTCH2
NTRK2
NOTCH2
NOTCH4
GLI3
WEE1
SPEN
COL1A1
23 24 31 32 33
1st Biopsy (0)
11 12 14 15 16 17 18 19 1A 1D 1G 1H
Tumour resection (6)
b
CB1004
WES
21 22 23 31
Diagnosis (0)
11 12 13 14 15 16 17 18 1A 1B 1C 1E
Tumour resection (5)
c
CB1005
WES
21
Diag.
11 12 13 14 17 1C 1D 1G 1H
Tumour resection (4)
d
CB1006
WES
11 12 13 14
Diagnosis (0)
e
CB1007
WES
11 12 13
Diagnosis (0)
VAF %
1
0.5
0

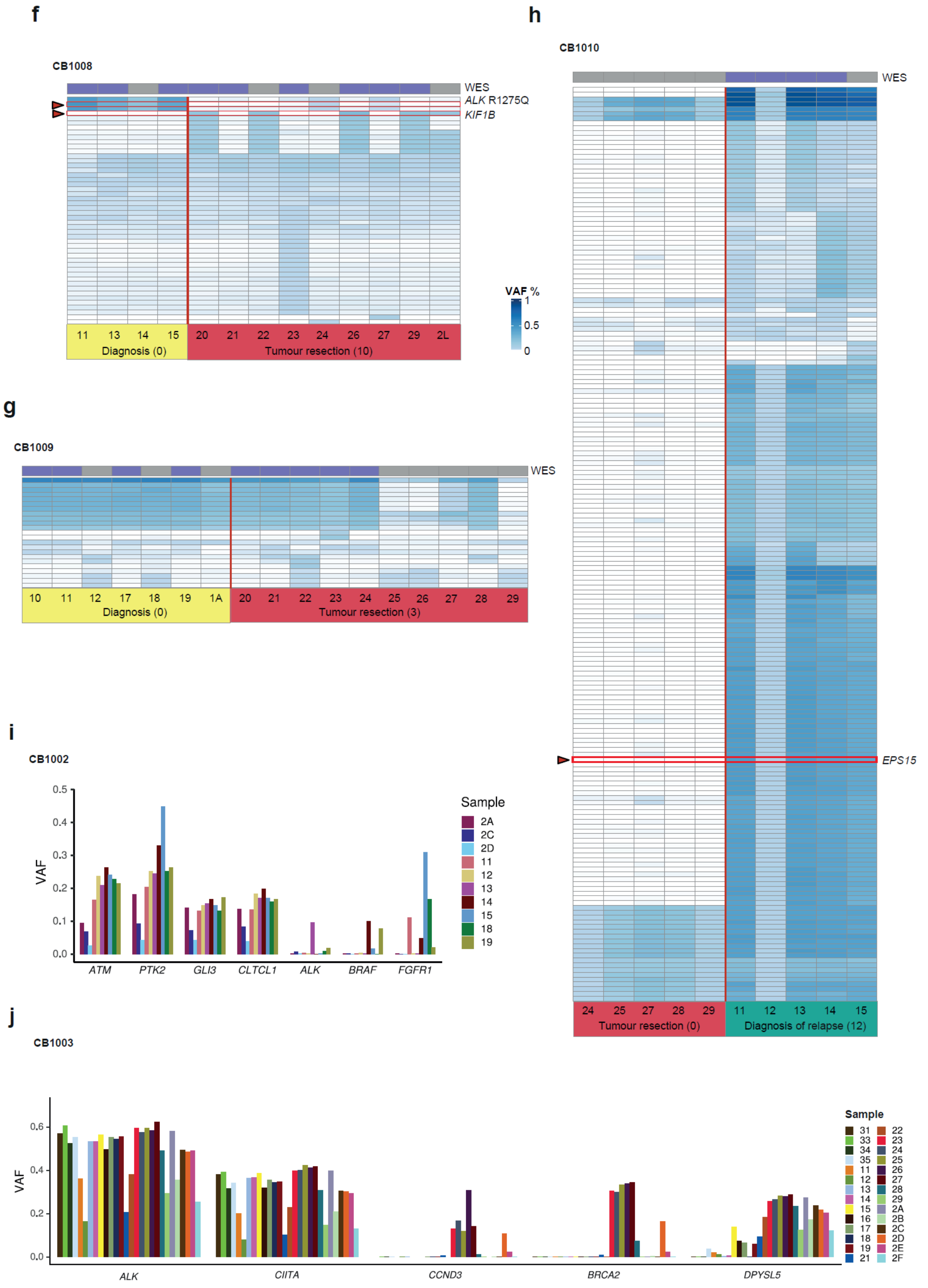

Supplementary Figure 2: Intratumour heterogeneity of SNVs analysed by ultra-deep targeted sequencing across the cohort. Variant allele frequency of somatic SNVs based on targeted sequencing data in patient CB1001 (**a**), patient CB1004 (**b**), patient CB1005 (**c**), patient CB1006 (**d**), patient CB1007 (**e**), patient CB1008 (**f**), patient CB1009 (**g**) and patient CB1010 (**h**). Only SNVs detected in one or more samples are displayed as one column per

SNV with a blue color code representing the percentage of their VAF. Mutations in cancer-related genes are named. Cancer-related genes with a clonal presence in a tumour are marked (green triangles) in comparison to subclonal oncogenic mutations (red triangles). SNVs detected at separate positions of a gene are listed twice. Row numbers state the sample number, and coloring indicates time of sample collection. Time (in month) elapsed from the initial diagnosis of neuroblastoma is indicated in parentheses. Columns to the right of the heatmap indicate further sample characteristics. "WES" indicates samples already included in the exome sequencing data. **i + j** Barplots represent the VAF (%) of cancer related genes in all samples analyzed by targeted sequencing from patient CB1002 (i) and CB1003 (j) as depicted in Fig. 2b + c.

a

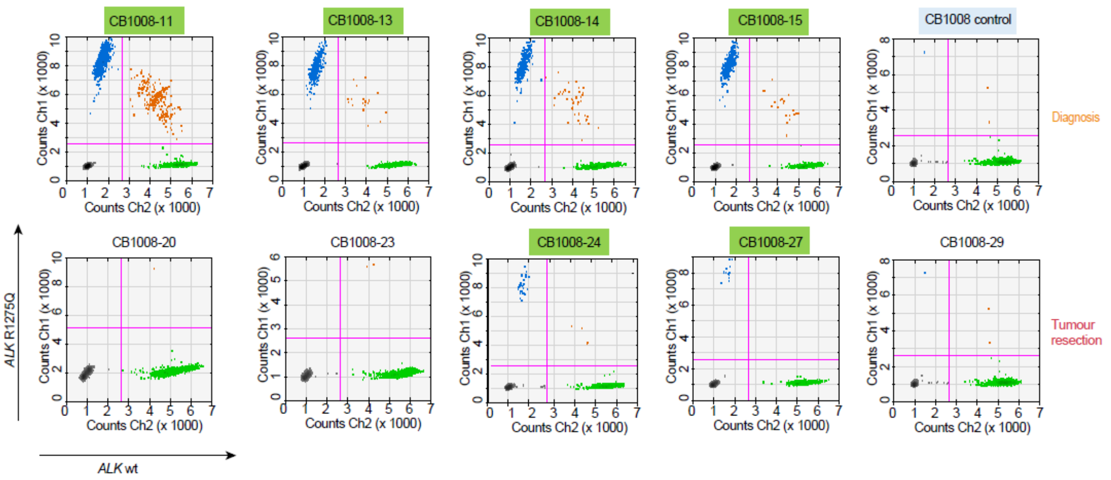

b

Sample	ddPCR (% VAF)	*ALK* R1275Q +/-	Targeted Seq (% VAF)	*ALK* R1275Q +/-
CB1008-11	47.4 ± 0.7	+	46.9	+
CB1008-13	38.5 ± 0.7	+	30.4	+
CB1008-14	22.1 ± 0.6	+	18.7	+
CB1008-15	41.1 ± 1.8	+	37.5	+
CB1008-20	0	-	0	-
CB1008-21	0	-	0	-
CB1008-22	0	-	0	-
CB1008-23	0	-	0	-
CB1008-24	2.1 ± 0.1	+	2.9	+
CB1008-26	0	-	0	-
CB1008-27	1.5 ± 0.3	+	1.1	+
CB1008-29	0	-	0	-
CB1008-2L	0	-	0	-
CB1008 control	0	-	0	-

Supplementary Figure 3: Validation of the temporal heterogeneity of the *ALK* R1275Q mutation in samples from CB1008 using digital droplet PCR (ddPCR). a Representative two-dimensional plots of fluorescent droplet counts after ddPCR of initial biopsies (CB1008-11 to -15) and the germline control (control) (first row) and of tumour resection samples (CB1008-20 to -29) (second row) from patient CB1008. *ALK* R1275Q droplets were plotted in channel 1 (Ch1) against *ALK* wildtype droplets in channel 2 (Ch2). Lines indicate thresholds for negative as well as single- and double-positive droplets. Blue dots (channel 1) and orange dots (channel 2) indicate *ALK* R1275Q positive droplets; green dots represent *ALK* wt droplets; gray dots indicate negative droplets. **b** VAF based on ddPCR assay from CB1008 samples confirmed results from targeted re-sequencing. Samples harbouring the *ALK* R1275Q are marked by green shading. A blood sample (blue shading) was used as control. Mean VAF (%) +/- SD from 3 independent measurements are shown.

a

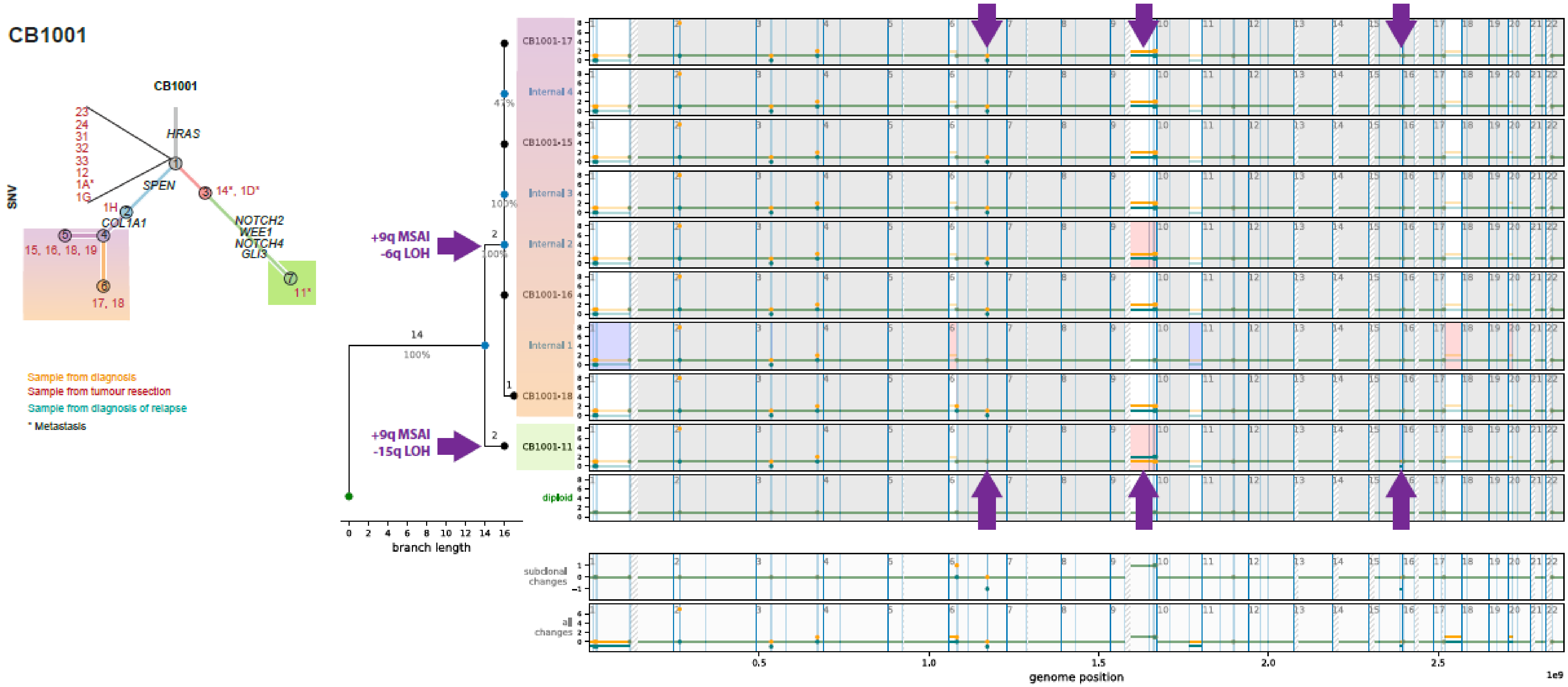

CB1001
CB1001
HRAS
SPEN
COL1A1
NOTCH2
WEE1
NOTCH4
GLI3
SNV
23
24
31
32
33
12
1A*
1G
1H
14*, 1D*
15, 16, 18, 19
17, 18
11*
Sample from diagnosis
Sample from tumour resection
Sample from diagnosis of relapse
* Metastasis
+9q MSAI
-6q LOH
+9q MSAI
-15q LOH
branch length
CB1001-17
Internal 4
CB1001-15
Internal 3
Internal 2
CB1001-16
Internal 1
CB1001-18
CB1001-11
diploid
subclonal changes
all changes
genome position

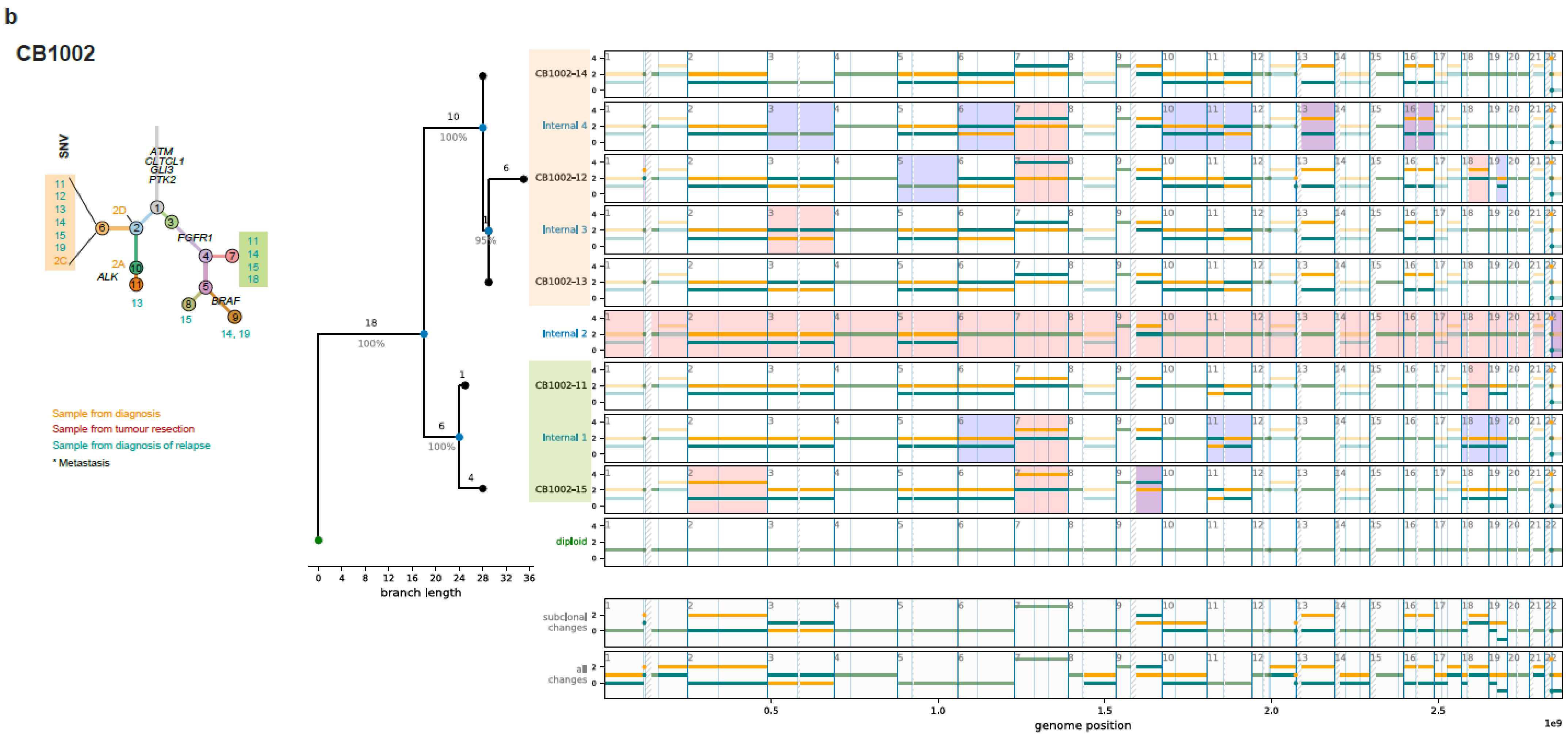
b
CB1002
SNV
ATM
CLTCL1
GLI3
PTK2
FGFR1
ALK
BRAF
2D
2A
2C
11
12
13
14
15
19
18
14, 19
Sample from diagnosis
Sample from tumour resection
Sample from diagnosis of relapse
* Metastasis
100%
95%
branch length
CB1002-14
Internal 4
CB1002-12
Internal 3
CB1002-13
Internal 2
CB1002-11
Internal 1
CB1002-15
diploid
subclonal changes
all changes
genome position
1e9

c

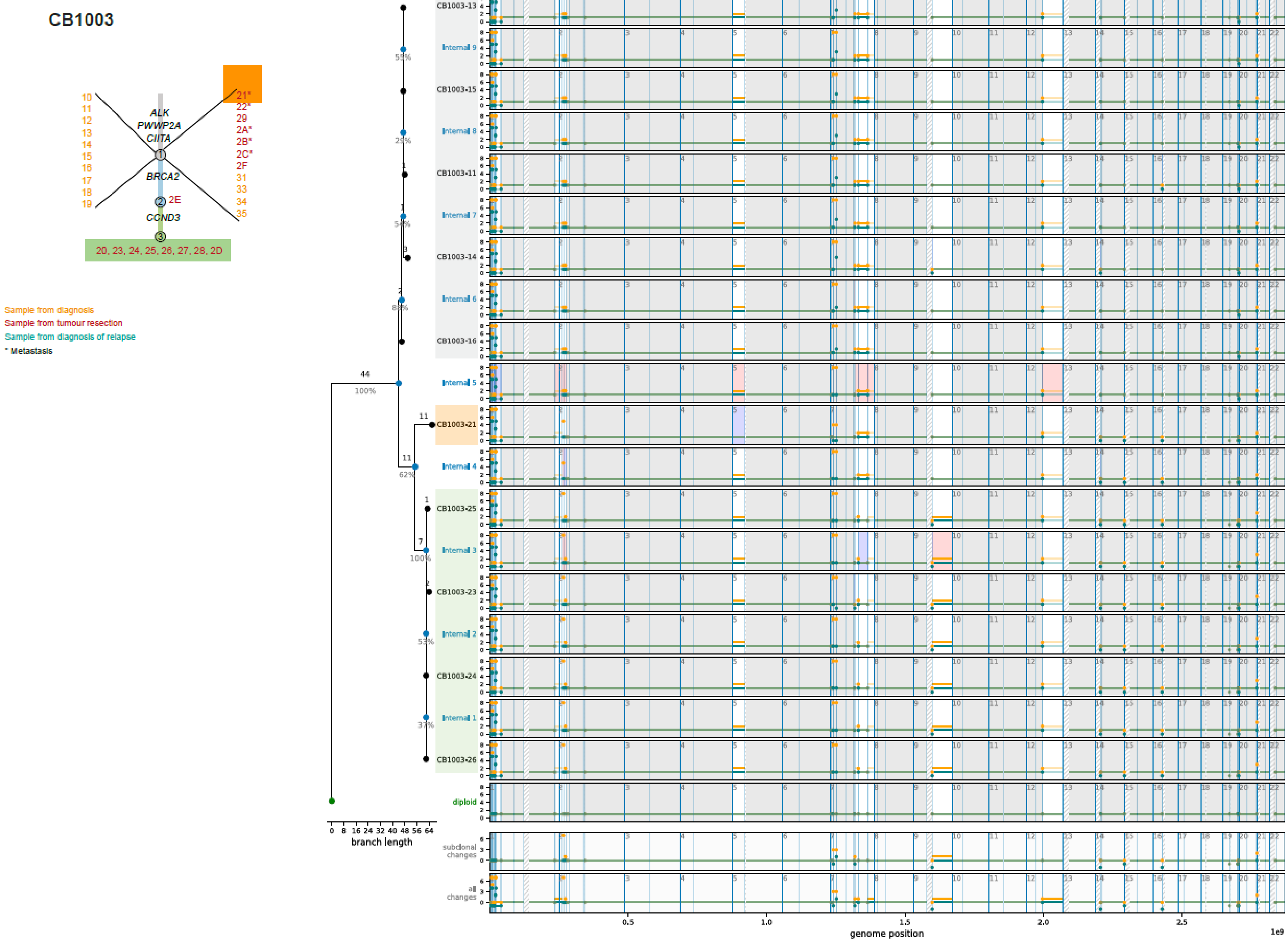
CB1003
ALK
PWWP2A
CIITA
BRCA2
CCND3
10
11
12
13
14
15
16
17
18
19
21*
22*
29
2A*
2B*
2C*
2F
31
33
34
35
2E
20, 23, 24, 25, 26, 27, 28, 2D
Sample from diagnosis
Sample from tumour resection
Sample from diagnosis of relapse
* Metastasis
CB1003-13
Internal 9
CB1003-15
Internal 8
CB1003-11
Internal 7
CB1003-14
Internal 6
CB1003-16
Internal 5
CB1003-21
Internal 4
CB1003-25
Internal 3
CB1003-23
Internal 2
CB1003-24
Internal 1
CB1003-26
diploid
subclonal changes
all changes
44
100%
11
62%
7
100%
branch length
0 8 16 24 32 40 48 56 64
genome position
0.5
1.0
1.5
2.0
2.5
1e9

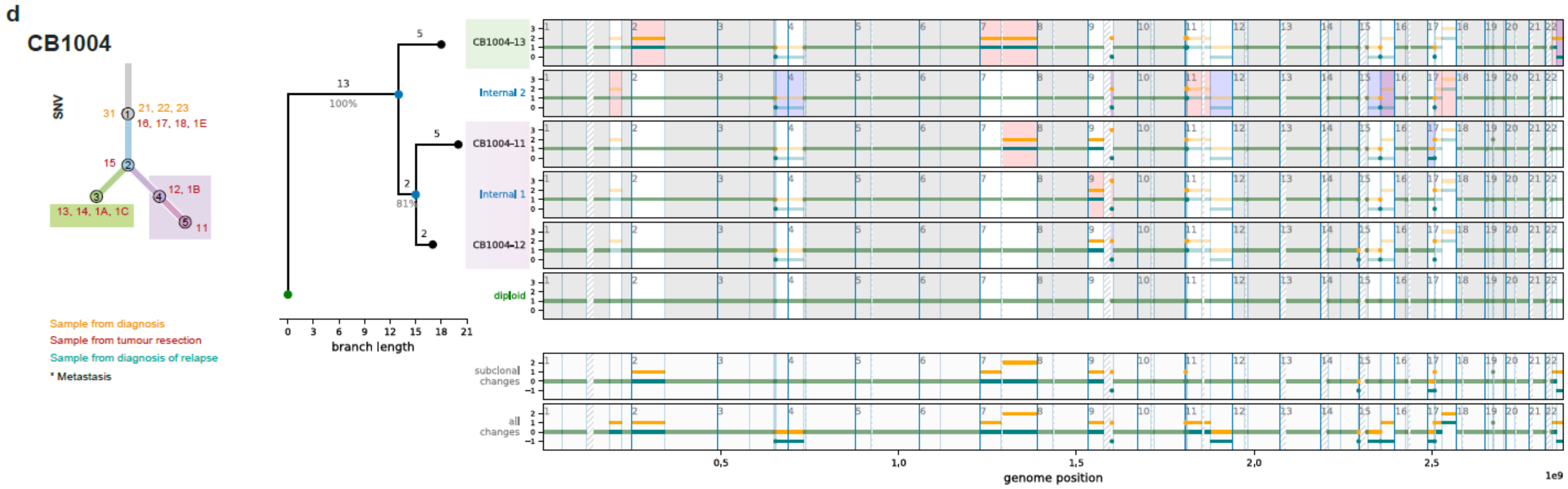

d
CB1004
SNV
31
21, 22, 23
16, 17, 18, 1E
15
12, 1B
13, 14, 1A, 1C
11
Sample from diagnosis
Sample from tumour resection
Sample from diagnosis of relapse
* Metastasis
13
100%
5
2
81%
branch length
CB1004-13
Internal 2
CB1004-11
Internal 1
CB1004-12
diploid
subclonal changes
all changes
genome position
1e9

e

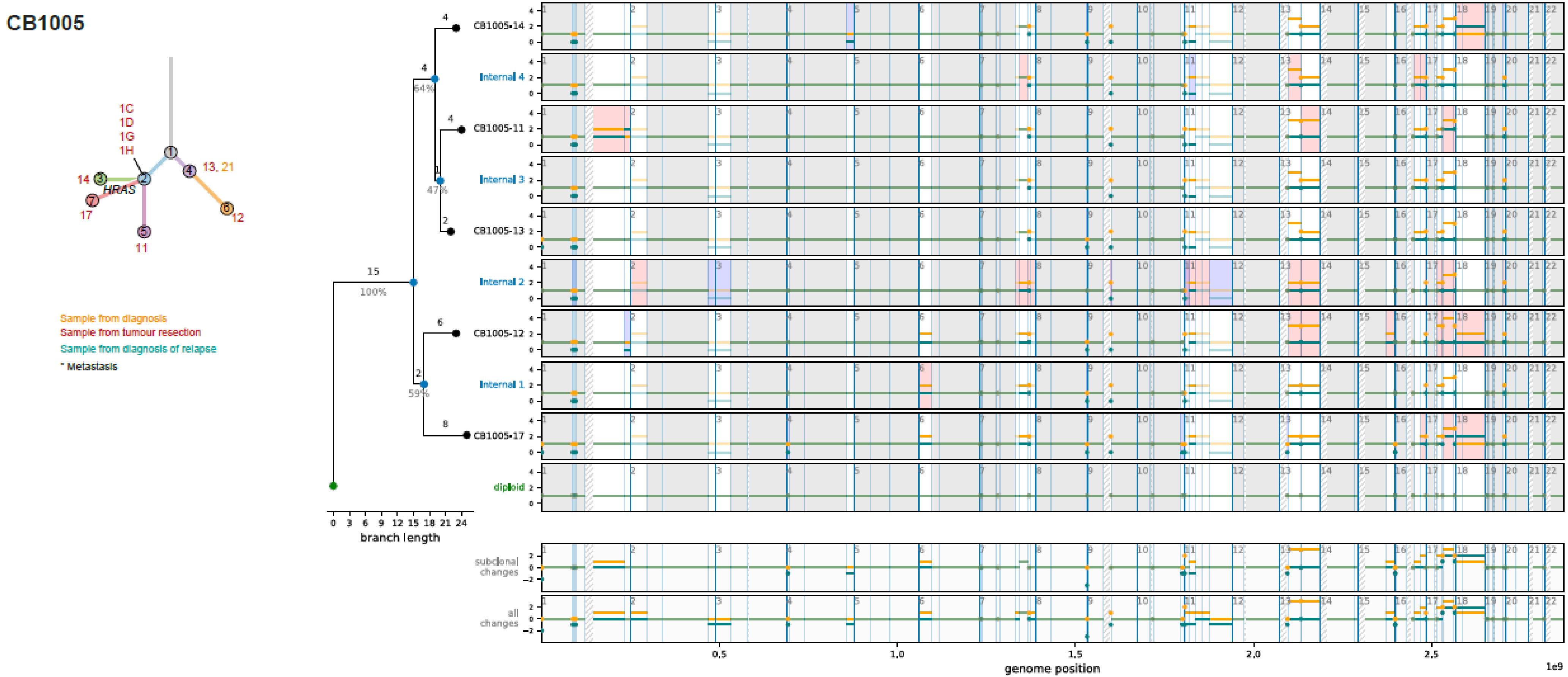
CB1005
1C
1D
1G
1H
HRAS
13, 21
14
17
11
12
Sample from diagnosis
Sample from tumour resection
Sample from diagnosis of relapse
* Metastasis
100%
64%
47%
59%
branch length
CB1005-14
Internal 4
CB1005-11
Internal 3
CB1005-13
Internal 2
CB1005-12
Internal 1
CB1005-17
diploid
subclonal changes
all changes
genome position
1e9

f

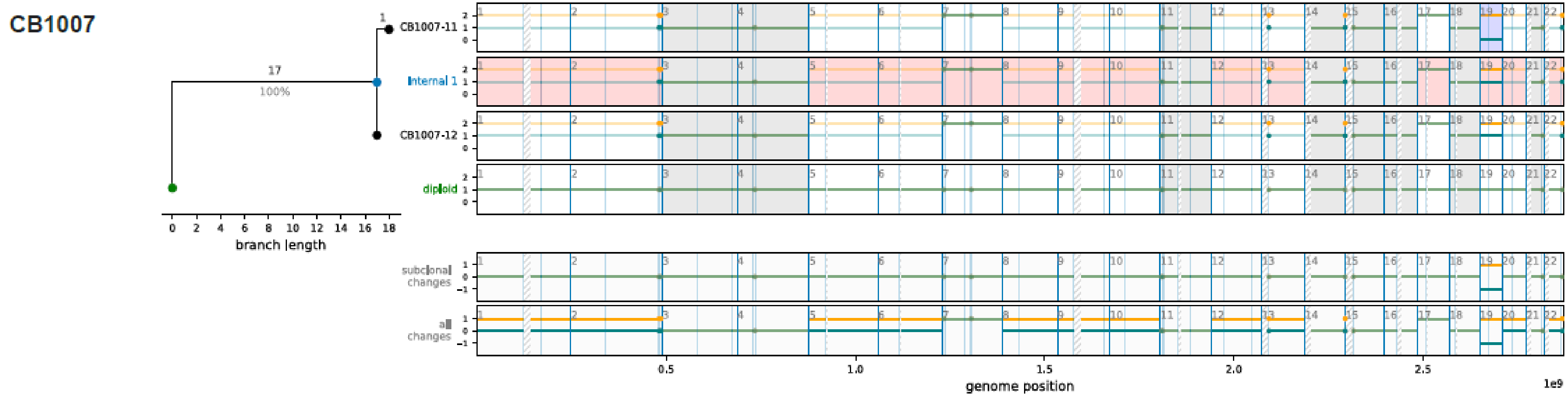

CB1007
17
100%
1
CB1007-11
Internal 1
CB1007-12
diploid
branch length
subclonal changes
all changes
genome position
1e9

g

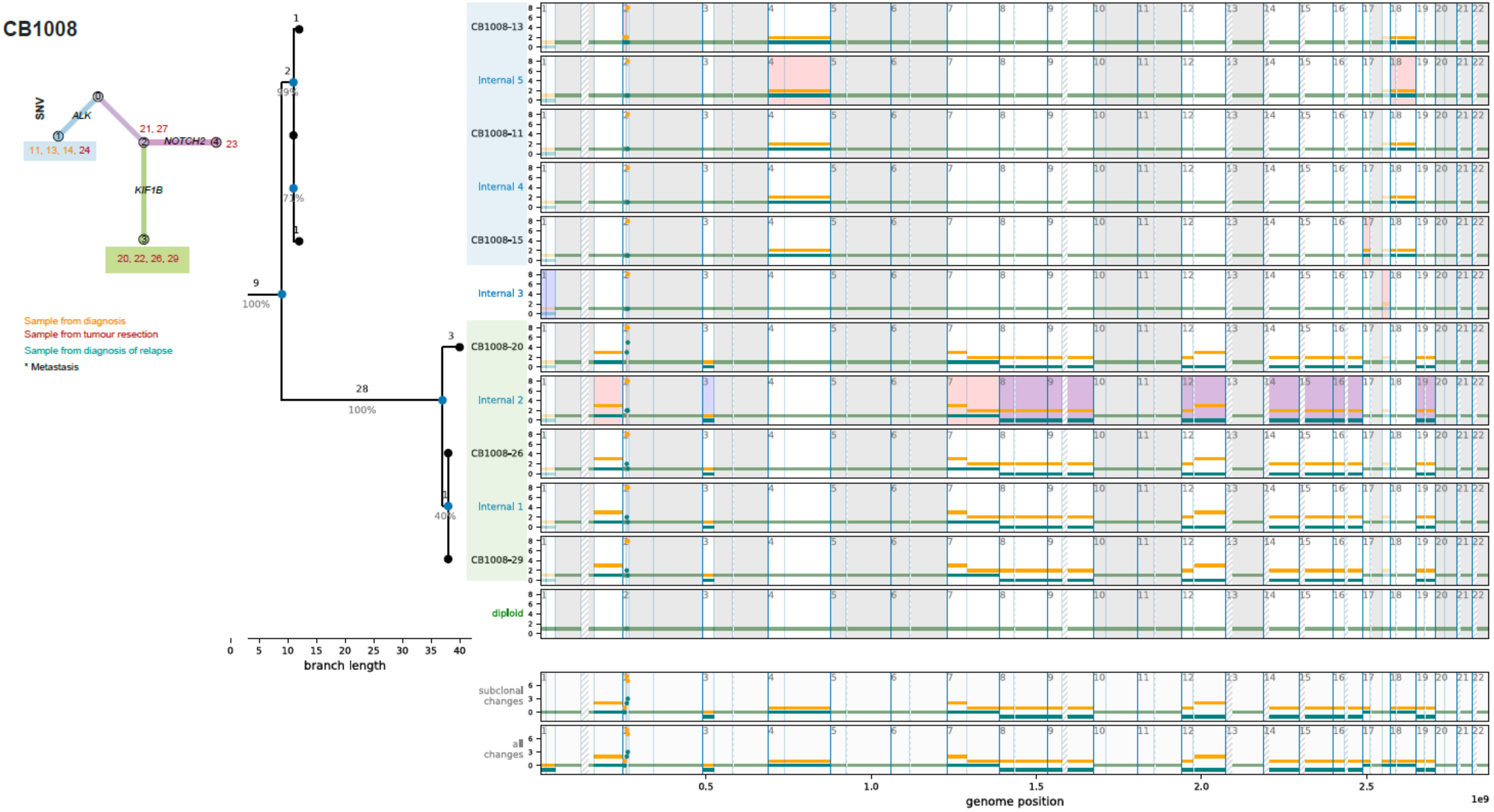
CB1008
SNV
ALK
NOTCH2
KIF1B
11, 13, 14, 24
21, 27
23
20, 22, 26, 29
Sample from diagnosis
Sample from tumour resection
Sample from diagnosis of relapse
* Metastasis
branch length
CB1008-13
Internal 5
CB1008-11
Internal 4
CB1008-15
Internal 3
CB1008-20
Internal 2
CB1008-26
Internal 1
CB1008-29
diploid
subclonal changes
all changes
genome position
1e9

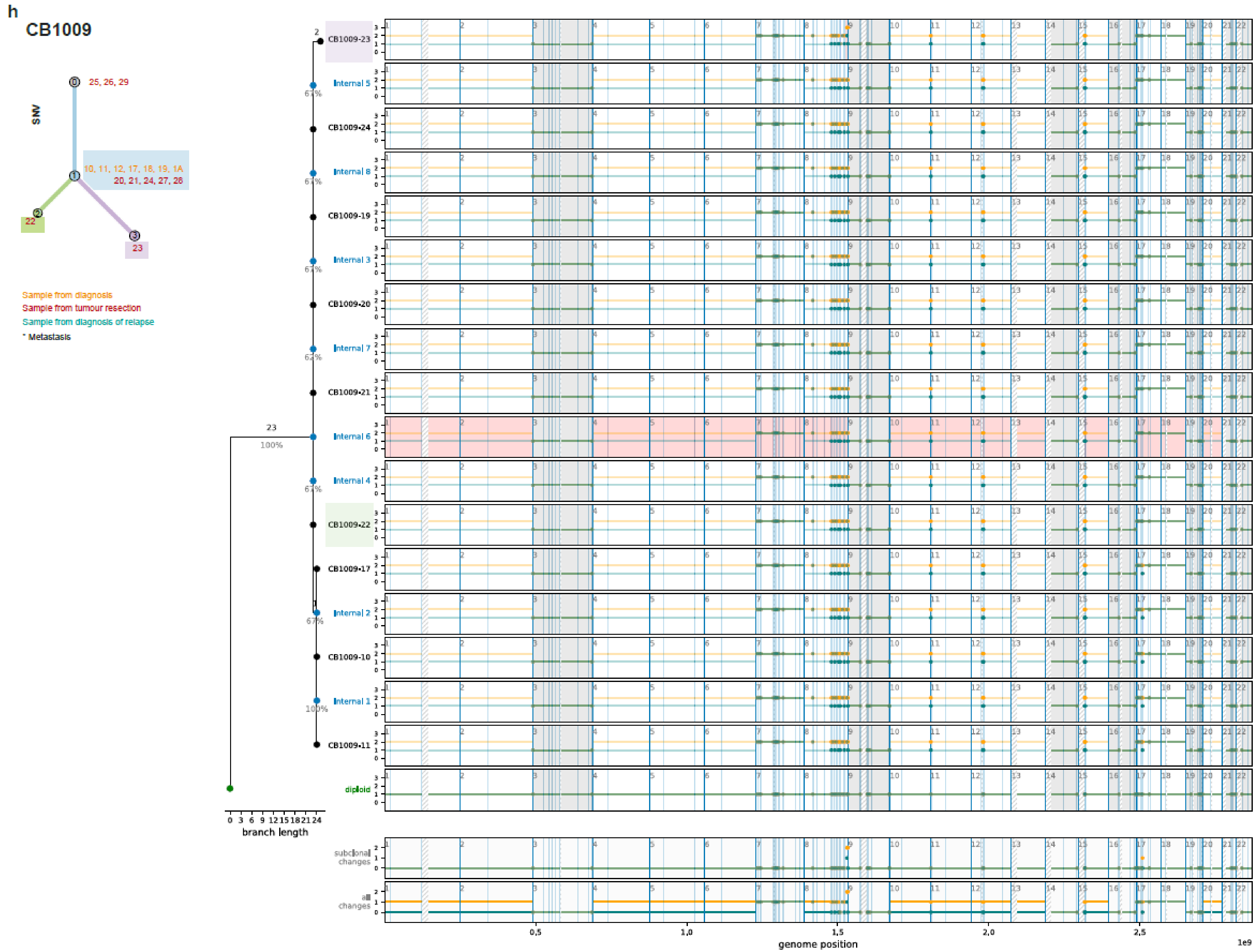
h
CB1009
SNV
25, 26, 29
10, 11, 12, 17, 18, 19, 1A
20, 21, 24, 27, 28
22
23
Sample from diagnosis
Sample from tumour resection
Sample from diagnosis of relapse
* Metastasis
CB1009-23
Internal 5
CB1009-24
Internal 8
CB1009-19
Internal 3
CB1009-20
Internal 7
CB1009-21
Internal 6
Internal 4
CB1009-22
CB1009-17
Internal 2
CB1009-10
Internal 1
CB1009-11
diploid
100%
branch length
subclonal changes
all changes
genome position
1e9

i

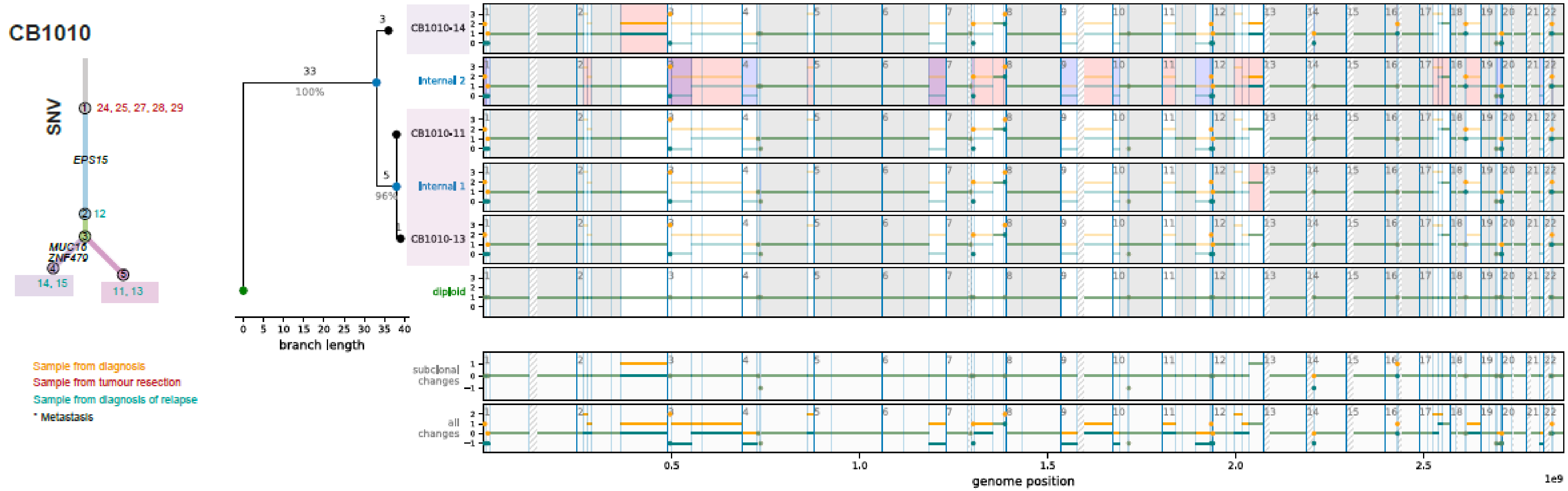

Supplementary Figure 4: Comparison of phylogenetic evolution based on SNVs and SCNAs. Phylogenetic trees based on SNVs (left side) and SCNAs (right side) were constructed with branch length corresponding to the number of detected variations. For SNV trees, sample IDs at the end of each branch indicate samples containing all clones (numbers in circles) of the branch and its ancestor clones. Samples were collected at diagnosis (orange), at tumour resection (red), at relapse diagnosis (turquoise). Metastatic samples are indicated by asterisks. For comparison, corresponding samples in the SNV tree and in the SCNA tree are highlighted with colored rectangles. Right-hand side shows copy-number states along the genome for both haplotypes (orange, green) for all sampled terminal nodes (black) and inferred ancestral (internal) nodes (blue) of the phylogenetic tree. Grey segments indicate no change compared to the diploid, red and blue segments indicate the occurrence of gain and loss events, respectively. The two bottom tracks display the net copy-number changes along the genome for subclonal (top) and all (bottom) evolutionary events.

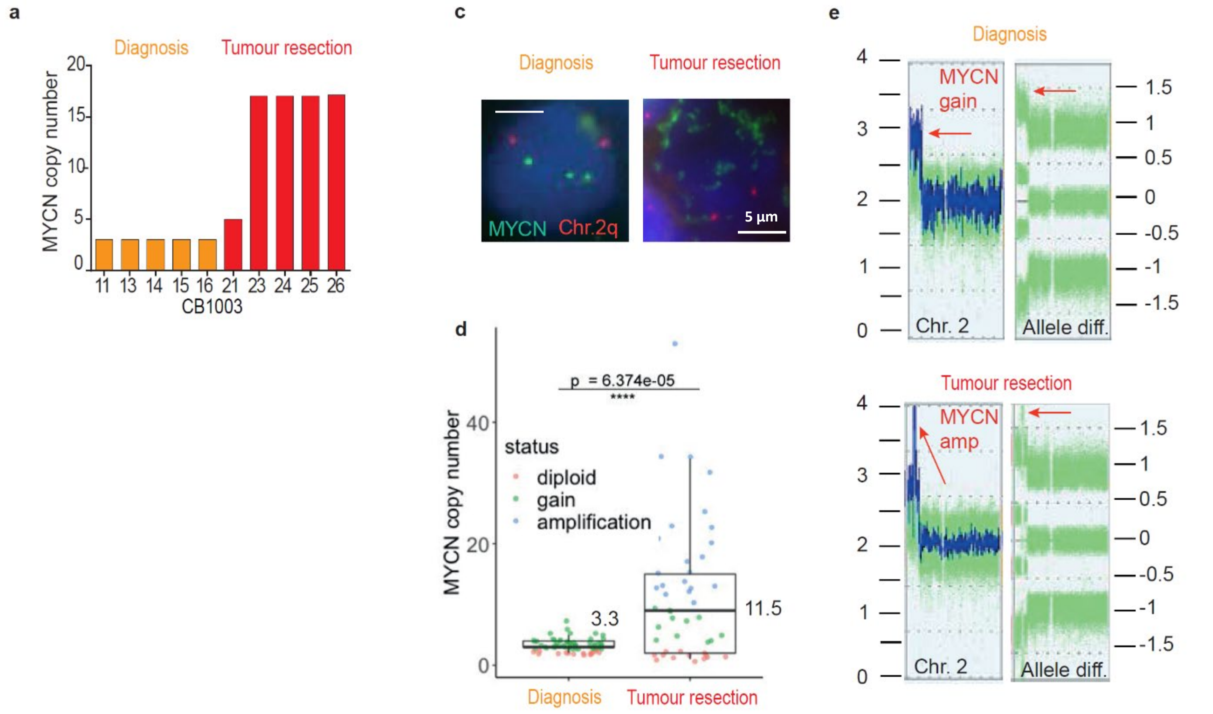

Supplementary Figure 5: *MYCN* FISH analysis and SNP array confirm temporal ITH of MNA in samples from patient CB1003. a *MYCN* copy numbers calculated based on WES. Samples collected as biopsy at diagnosis had no MNA, while 8 month later, a MNA was detected in four samples of the primary tumour resection (23-26) but not in the lymph node metastasis resected at the same time (21). **c** *MYCN* FISH analysis using probes for *MYCN* (green) and 2q23 as reference (red, *NMI* gene region) in samples from patient CB1003. Samples at diagnosis with a 2p gain (left panel) and samples from the tumour resection (right panel) with double minutes are shown. **d** *MYCN* FISH signals were quantified as a ratio of *MYCN*/reference signals using *ImageJ* software. *MYCN* copy number was defined as amplified for a ratio of > 9 compared to the reference and was displayed as median for n=34 and n=82 cells (diagnosis and tumour resection, respectively). Data are represented as boxplots with middle line as median, lower and upper hinges correspond to the first and third quartiles, whiskers extend from the hinges to the largest value no further than 1.5 × inter-quartile range. Statistical significance was calculated using two-sided Mann-Whitney test. **e** Detection of the MNA in tumour resection samples (lower panel) versus diagnostic samples (upper panel) by SNP array from DNA isolated from tumour bulk. Copy numbers are visualized in exemplary samples using the smoothed signal (left) and the allele difference plot (right).

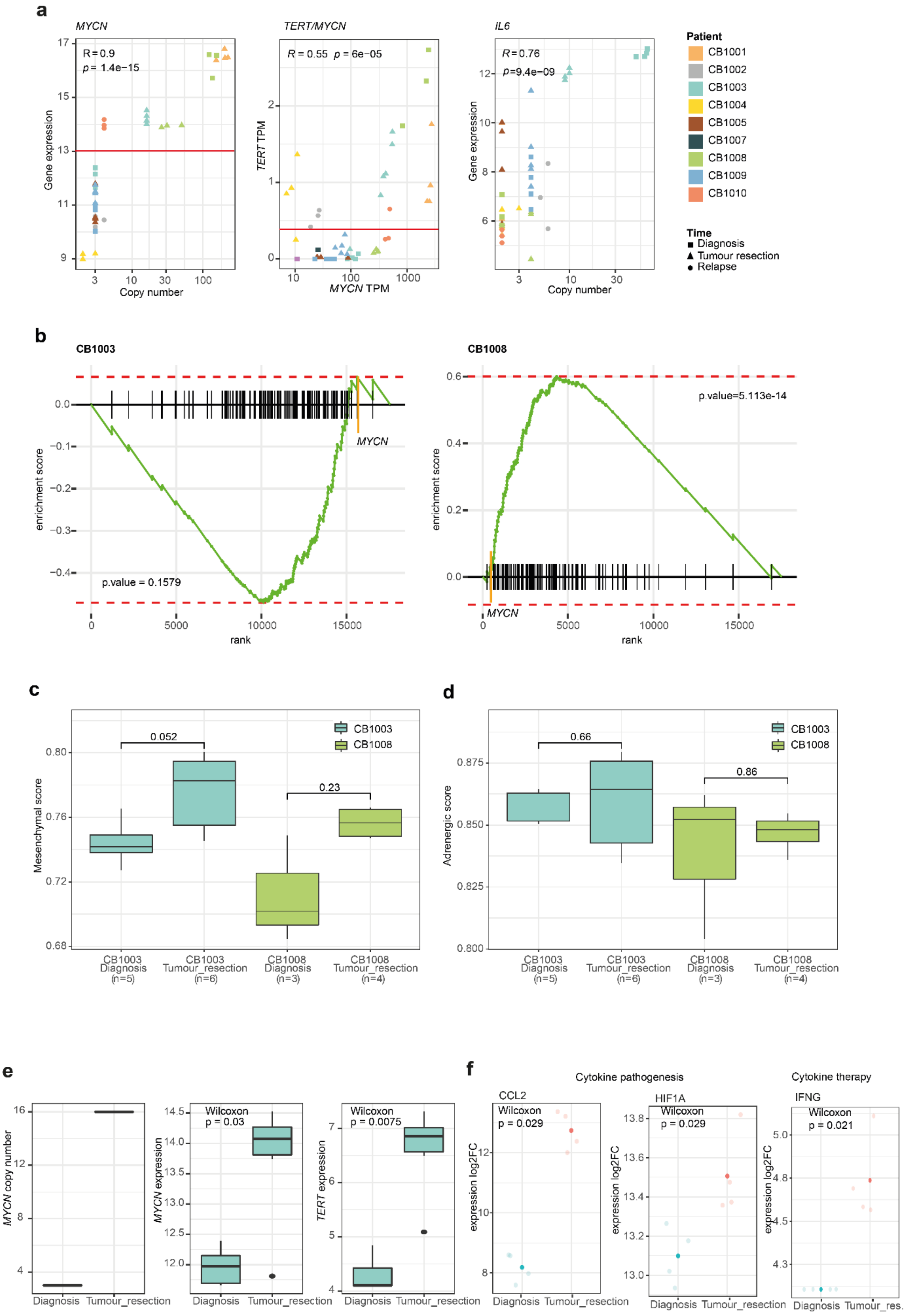
a
MYCN
R = 0.9
p = 1.4e−15
Gene expression
Copy number
TERT/MYCN
R = 0.55 p = 6e−05
TERT TPM
MYCN TPM
IL6
R = 0.76
p = 9.4e−09
Patient
CB1001
CB1002
CB1003
CB1004
CB1005
CB1007
CB1008
CB1009
CB1010
Time
Diagnosis
Tumour resection
Relapse
b
CB1003
enrichment score
p.value = 0.1579
MYCN
rank
CB1008
p.value=5.113e-14
c
0.052
0.23
Mesenchymal score
CB1003 Diagnosis (n=5)
CB1003 Tumour_resection (n=6)
CB1008 Diagnosis (n=3)
CB1008 Tumour_resection (n=4)
d
0.66
0.86
Adrenergic score
e
MYCN copy number
Diagnosis
Tumour_resection
Wilcoxon
p = 0.03
MYCN expression
Wilcoxon
p = 0.0075
TERT expression
f
Cytokine pathogenesis
Cytokine therapy
CCL2
Wilcoxon
p = 0.029
expression log2FC
HIF1A
Wilcoxon
p = 0.029
IFNG
Wilcoxon
p = 0.021
Tumour_res.

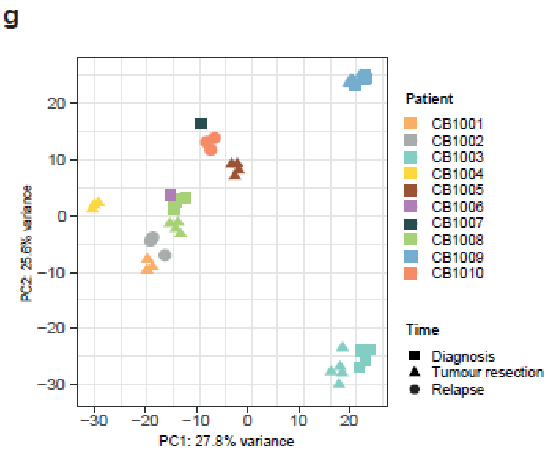

Supplementary Figure 6: Temporal and spatial changes in RNA expression in consequence of SCNA assessed by multi-region RNA sequencing. a Copy number driven expression is depicted for *MYCN* (left panel) and *IL6* (right panel) in correlation to gene locus copy number for all 10 neuroblastoma patients. Expression of *TERT* in samples from patients with high *MYCN* expression, or independent from *MYCN* copies (CB1001) (middle panel). *P*-values were calculated using the two-sided Pearson's correlation coefficient. **b** Gene set enrichment analysis (GSEA) for *MYCN* dependent genes (s. Supplementary Data 2) for samples from diagnostic versus tumour resection from patients CB1003 and CB1008. *P*-values are estimated from enrichment score significance according to *R package fgsea* 1.16.0. **c+d** Diagnostic versus tumour resection samples from patients CB1003 and CB1008 were analysed for differentiation-related expression profiles comprising mesenchymal and adrenergic signatures calculated as in (Groningen et al.)[6]. **e** The acquisition of the *MYCN* amplification and the increase of *MYCN* expression in tumour resection samples from CB1003. **f** Temporal expression changes of exemplary genes in samples from CB1003 at both time points. **g** Principal-component analysis (PCA) based on the 100 most highly variable genes (see Supplementary Data 4) in all samples shows homogenous expression profiles within each patient. **c+d, e** Data are represented as boxplots with middle line as median, lower and upper hinges correspond to the first and third quartiles, whiskers extend from the hinges to the largest value no further than 1.5 × inter-quartile range.

Supplementary Tables

Supplementary Table 1: Patients characteristics

Patient ID	*MYCN* Amp	*ALK*-Mut.	Diagnosis	DOD	Stage	Risk group
CB1001	yes	no	NB	yes	III	HR
CB1002	no	no	NB	yes	IV	HR
CB1003	later	yes	NB	yes	IV	HR
CB1004	no	no	NB	no	IV	HR
CB1005	no	no	NB	no	IV	HR
CB1006	no	no	NB	no	II	LR
CB1007	no	no	NB	no	II	LR
CB1008	yes	yes	NB	no	IV	HR
CB1009	no	no	NB	no	III	IMR
CB1010	no	no	NB	yes	IV	HR

Abbreviations: NB: neuroblastoma, DOD: death of disease, HR: high risk, IMR: intermediate risk, LR: low risk,

Supplementary Table 2: SNVs in cancer-related genes and their biological relevance

						Mutation		Gene listed in			Mutation	
**Symbol**	**RefSeq**	**Description**	**Location**	**Mutation**		**recurrent**	**pathogenic**	**Cosmic tier 1**	**Gröbner et al. [4]**	**Worst et al. [5]**	**NB related as in**	**expressed**
**ALK**	**NM_001353765**	**ALK receptor tyrosine kinase**	**2p23.2-p23.1**	**F1174L**		**1**	**1**	**1**	**1**	**1**	**1, 2, 3**	**1**
**ALK**	**NM_001353765**	**ALK receptor tyrosine kinase**	**2p23.2-p23.1**	**R1275Q**		**1**	**1**	**1**	**1**	**1**	**1, 2, 3**	**1**
ATM	NM_000051	ATM serine/threonine kinase	11q22.3	G1307W		0	0	1	0	1		
**BRAF**	**NM_001354609**	**B-Raf proto-oncogene, serine/threonine kinase**	**7q34**	**V600E**		**1**	**1**	**1**	**1**	**1**	**2, 3**	**partially**
BRCA2	NM_000059	BRCA2 DNA repair associated	13q13.1	regulatory	c.-4776G>T	0	0	1	1	1		
BRCA2	NM_000059	BRCA2 DNA repair associated	13q13.1	regulatory	c.-4775G>T	0	0	1	1	1		
CCND3	NM_001760.4	cyclin D3	6p21.1	regulatory	c.*3520C>G	0	0	1	1	1		
CIITA	NM_000246	class II major histocompatibility complex transactivator	16p13.13	P831H		0	0	1	0	0		
CLTCL1	NM_001835	clathrin heavy chain like 1	22q11.21	G290S		0	0	1	0	0		
COL1A1	NM_000088	collagen type I alpha 1 chain	17q21.33	G569V		0	0	1	1	0		
EPS15	NM_001159969	epidermal growth factor receptor pathway substrate 15	1p32.3	D399V		0	1	1	0	0		
**FGFR1**	**NM_001174063**	**fibroblast growth factor receptor 1**	**8p11.23**	**N577K**		**1**	**1**	**1**	**1**	**1**	**2, 3**	**partially**
GLI3	NM_000168	GLI family zinc finger 3	7p14.1	V891M		0	?	0	0	1		
GLI3	NM_000168	GLI family zinc finger 3	7p14.1	P195L		0	?	0	0	1		
**HRAS**	**NM_001130442**	**HRas proto-oncogene, GTPase**	**11p15.5**	**Q61K**		**1**	**1**	**1**	**0**	**1**	**2**	**1**
KCNJ5	NM_000890	potassium inwardly rectifying channel subfamily	11q24.3	L386M		0	0	1	0	0		
KIF1B	NM_001365951	kinesin family member 1B	1p36.22	R1436Q		0	0	0	0	1		
NOTCH2	NM_001200001	notch receptor 2	1p12	P2387H		0	1	1	1	1		
NOTCH4	NM_004557	notch receptor 4	6p21.32	G246E		0	?	0	0	1		
PTK2	NM_001199649	protein tyrosine kinase 2	8q24.3	N361S		0	?	0	0	1		
SPEN	NM_015001	spen family transcriptional repressor	1p36.21-p36.13	R2084T		0	0	1	0	0		
WEE1	NM_001143976	WEE1 G2 checkpoint kinase	11p15.4	F179L		0	0	1	0	1		

Supplementary References

1. Pugh, T.J. *et al.* The genetic landscape of high-risk neuroblastoma. *Nature Genetics* **45**, 279-284 (2013).
2. Ackermann, S. et al. A mechanistic classification of clinical phenotypes in neuroblastoma. Science (New York, N.Y.) 362, 1165-1170 (2018).
3. Brady, S.W. *et al.* Pan-neuroblastoma analysis reveals age- and signature-associated driver alterations. *Nat Commun* **11**, 5183 (2020).
4. Gröbner, S.N. et al. The landscape of genomic alterations across childhood cancers. Nature 555, 321-327 (2018).
5. Worst, B.C. et al. Next-generation personalized medicine for high-risk paediatric cancer patients - The INFORM pilo study. EJC 65, 91-101 (2016)
6. van Groningen, T. *et al.* Neuroblastoma is composed of two super-enhancer-associated differentiation states. *Nat Genet* **49**, 1261-1266 (2017).
