# Supplementary material for: Spatial and temporal intratumour heterogeneity has potential consequences for single biopsy-based neuroblastoma treatment decisions
Source: Nat Commun. 2021 Nov 23;12:6804. doi: 10.1038/s41467-021-26870-z (PMC8611017; doi:10.1038/s41467-021-26870-z)
Supplement: Supplementary file 3 — Description of Additional Supplementary Files [file 41467_2021_26870_MOESM3_ESM.pdf]

Description of Additional Supplementary Files

File Name: Supplementary Data 1
Description: List of 801 genes considered to be cancer-related according to COSMIC and literature as described in the methods section.

File Name: Supplementary Data 2:
Description: List of published MYCN target genes.

File Name: Supplementary Data 3:
Description: List of differentially expressed genes of high-risk neuroblastoma.

File Name: Supplementary Data 4:
Description: List of the 100 most variable expressed genes assessed by RNA Seq in our 10 patient neuroblastoma cohort.

File Name: Supplementary Software 1
Description: Source code in *R* for running *sciClone* and for constructing the clonality trees provided

as a zipped file.
