# Supplementary material for: Spatial and temporal intratumour heterogeneity has potential consequences for single biopsy-based neuroblastoma treatment decisions
Source: Nat Commun. 2021 Nov 23;12:6804. doi: 10.1038/s41467-021-26870-z (PMC8611017; doi:10.1038/s41467-021-26870-z)
Supplement: Supplementary file 9 — Reporting Summary [file 41467_2021_26870_MOESM9_ESM.pdf]

nature research

Corresponding author(s): Prof. Angelika Eggert

Last updated by author(s): Sep 1, 2021

Reporting Summary

Nature Research wishes to improve the reproducibility of the work that we publish. This form provides structure for consistency and transparency in reporting. For further information on Nature Research policies, see our Editorial Policies and the Editorial Policy Checklist.

Statistics

For all statistical analyses, confirm that the following items are present in the figure legend, table legend, main text, or Methods section.

n/a	Confirmed	
☐	☒	The exact sample size (*n*) for each experimental group/condition, given as a discrete number and unit of measurement
☒	☐	A statement on whether measurements were taken from distinct samples or whether the same sample was measured repeatedly
☐	☒	The statistical test(s) used AND whether they are one- or two-sided<br>*Only common tests should be described solely by name; describe more complex techniques in the Methods section.*
☒	☐	A description of all covariates tested
☒	☐	A description of any assumptions or corrections, such as tests of normality and adjustment for multiple comparisons
☐	☒	A full description of the statistical parameters including central tendency (e.g. means) or other basic estimates (e.g. regression coefficient) AND variation (e.g. standard deviation) or associated estimates of uncertainty (e.g. confidence intervals)
☐	☒	For null hypothesis testing, the test statistic (e.g. *F*, *t*, *r*) with confidence intervals, effect sizes, degrees of freedom and *P* value noted<br>*Give P values as exact values whenever suitable.*
☒	☐	For Bayesian analysis, information on the choice of priors and Markov chain Monte Carlo settings
☒	☐	For hierarchical and complex designs, identification of the appropriate level for tests and full reporting of outcomes
☒	☐	Estimates of effect sizes (e.g. Cohen's *d*, Pearson's *r*), indicating how they were calculated

Our web collection on statistics for biologists contains articles on many of the points above.

Software and code

Policy information about availability of computer code

Data collection	No software for data collection was used.
Data analysis	All software tools used for data analysis are indicated in the method section of the manuscript. We used bcl2fastq (2.16), FastQ (0.11.5), bwa (0.7.17) samblaster (0.1.24), bedtools (2.27.0), sambamba (0.6.8) , EBCall (2), snpEff (4.3t), sciClone (1.1.0), STAR (2.7.0a), Subread (1.6.5) , R (4.0.2) , Bioconductor (3.11) , samtools (1.9) , Sequenza (3.0.0), Gencode transcript annotation (26) Chromosome Analysis Suite (ChAS) (4.1.0.90), MEDICC (2), QuantaSoft Analysis (1.7.4.0917).

For manuscripts utilizing custom algorithms or software that are central to the research but not yet described in published literature, software must be made available to editors and reviewers. We strongly encourage code deposition in a community repository (e.g. GitHub). See the Nature Research guidelines for submitting code & software for further information.

Data

Policy information about availability of data

All manuscripts must include a data availability statement. This statement should provide the following information, where applicable:

- Accession codes, unique identifiers, or web links for publicly available datasets
- A list of figures that have associated raw data
- A description of any restrictions on data availability

Raw sequencing data used in this study are available in the European Genome phenome archive (www.ebi.ac.uk/ega/) under the accession number EGA S00001004735 [https://ega-archive.org/datasets/EGAD00001008020] (WES), EGAD00001008156 (targeted re-sequencing), EGAD00001008133 (RNA Seq) and EGAD00010002225 (SNP array). Download of all datasets are available by contacting the study affiliated Data Access Committee (DAC) via EGA. For WES analysis, NCBI dbSNP (https://www.ncbi.nlm.nih.gov/snp) and UCSC Genome Browser (https://genome.ucsc.edu/) were used to aggregate somatic mutations. For defining cancer-related genes, public data was downloaded from COSMIC database (https://cancer.sanger.ac.uk). For MES/ADRN differentiation subsets, analysis were done

based on datasets downloaded from GEO (www.ncbi.nlm.nih.gov/geo/) (GSE90805). For analysis of differential gene expression between high- and low-risk tumours, data sets were downloaded from GEO with accession GSE49711.

Field-specific reporting

Please select the one below that is the best fit for your research. If you are not sure, read the appropriate sections before making your selection.

☒ Life sciences ☐ Behavioural & social sciences ☐ Ecological, evolutionary & environmental sciences

For a reference copy of the document with all sections, see nature.com/documents/nr-reporting-summary-flat.pdf

Life sciences study design

All studies must disclose on these points even when the disclosure is negative.

Sample size	In total, 140 tumor samples were analysed from 10 patients. Sample size was defined by biological availability and sample quality of the material (tumour purity, vitality) during the collection time of 2014 - 2018.
Data exclusions	Are indicated in "methods". In detail: During alignment of the WES seq raw data, FastQC was used to filter out PCR duplicates and failed alignment of reads; for clonality analysis, 2 patients were excluded due to low number of SNVs to infer trees. During RNA Seq, 3 out of 51 samples were excluded due to low quality RNA.
Replication	WES data were validated by ultra-deep targeted re-sequencing; SNV were analysed for expression of variant alleles in RNA Seq; MYCN copy numbers were verified by FISH. WES, RNA Seq and FISH analysis were done once due to its cost intensity and limited availibility of patient's tumour material.
Randomization	Randomization was not applicable since we did not analyze different experimental groups. In addition, data analysis was not part of a clinical study with different treatment arms.
Blinding	Blinding was not applicable since no different experimental groups or treatment arms were included. Nevertheless, analysis was performed for pseudonymizes samples.

Reporting for specific materials, systems and methods

We require information from authors about some types of materials, experimental systems and methods used in many studies. Here, indicate whether each material, system or method listed is relevant to your study. If you are not sure if a list item applies to your research, read the appropriate section before selecting a response.

Materials & experimental systems

n/a	Involved in the study	
☒	☐	Antibodies
☒	☐	Eukaryotic cell lines
☒	☐	Palaeontology and archaeology
☒	☐	Animals and other organisms
☐	☒	Human research participants
☐	☒	Clinical data
☒	☐	Dual use research of concern

Methods

n/a	Involved in the study	
☒	☐	ChIP-seq
☒	☐	Flow cytometry
☒	☐	MRI-based neuroimaging

Human research participants

Policy information about studies involving human research participants

Population characteristics	Patient's characteristics are provided as Supplementary table in the manuscript
Recruitment	Tumour specimens with sufficient material left after diagnostic procedures and with sufficient quality (tumour purity, lack of necrosis) were collected from 10 neuroblastoma patients at the Charité, Berlin, Germany, between 2014 and 2018. These patients were treated according to the German neuroblastoma trial (NB2004) or the German Neuroblastoma Registry (NB Registry 2016) of the GPOH. Registry patients who started their treatment outside of Germany were treated with European standard of care treatment and not within an intention to treat clinical trial. Selecting bias may be present for high-risk neuroblastoma due to a possible overrepresentative presentation of high-risk cases to the Charité. This might be reflected by only 2 out of 10 low-risk neuroblastomas in our study. This has been discussed in the manuscript in the context of SCNA analysis as well as in the response to the reviewers in the context of expression analysis.
Ethics oversight	Institutional Review Board of the Medical Faculty, University of Cologne as the trial sponsor of the NB2004 and NB registry 2016. This information is given in the methods section of the manuscript.

Note that full information on the approval of the study protocol must also be provided in the manuscript.

Clinical data

Policy information about clinical studies

All manuscripts should comply with the ICMJE guidelines for publication of clinical research and a completed CONSORT checklist must be included with all submissions.

Clinical trial registration	NB2004-HR ClinicalTrials.gov Identifier: NCT03042429
Study protocol	The protocol is available under: https://www.kinderkrebsinfo.de/sites/kinderkrebsinfo/content/e1676/e9032/e68518/e206421/download7673/NB_2004_1.00_komprimiert__ger.pdf
Data collection	Specifics of data collection are defined and described in detail in the clincial trial protocol.
Outcomes	Primary (EFS) and secondary outcome measures are defined and described in detail in the clincial trial protocol.
